# Predictive Factors of Transient Urinary Incontinence Following Holmium Laser Enucleation of the Prostate (HoLEP): Single-Center Experience

**DOI:** 10.3390/medicina60091460

**Published:** 2024-09-06

**Authors:** Roxana Andra Coman, Thomas Bschleipfer, Nadim Al Hajjar, Bogdan Petrut

**Affiliations:** 1 Department of Urology, “Iuliu Hatieganu” University of Medicine and Pharmacy, 400012 Cluj-Napoca, Romania; dr.roxanacoman@yahoo.com (R.A.C.);; 2 Department of Urology, Medicover Hospital, 407062 Suceagu, Romania; 3 Clinic for Urology and Paediatric Urology, Regiomed Clinics Coburg, Ketschendorfer Straße 33, 96450 Coburg, Germany; 4 Department of Surgery, “Iuliu Hatieganu” University of Medicine and Pharmacy, 400012 Cluj-Napoca, Romania; 5 “Octavian Fodor” Regional Institute of Gastroenterology and Hepatology, 400394 Cluj-Napoca, Romania

**Keywords:** prostate, BPH, enucleation, HoLEP, incontinence, lasers

## Abstract

*Background and Objectives*: The aim of this study was to assess the predictive factors associated with transient urine incontinence (TUI) following holmium laser enucleation of the prostate (HoLEP). *Materials and Methods*: A retrospective analysis was conducted on a prospectively maintained database containing the first 149 consecutive HoLEP cases between June 2022 and December 2023. The study recorded several patient characteristics, and preoperative data such as IPSS score, total gland volume, preoperative catheterization, Qmax, and PVR volume were collected. During the operation, data on total operating time, enucleation time, morcellation time, and weight of enucleated tissue were recorded. Finally, postoperative data were also documented. TUI refers to a patient’s complaint of urine leakage, irrespective of type. Univariate and multivariate logistic regression analyses were performed to determine factors that predict TUI. *Results*: The study included 119 patients with BPH. Nineteen (15.96%) of them experienced postoperative TUI. Of those 19 patients, 15 (78.94%) recovered within three months from the date of the surgery. In the multivariate regression analysis, increased age (odds ratio [OR], 3.47; 95% confidence interval [CI], 1.56~7.78; *p* = 0.002), prostate volume ≥ 100 mL (OR 1.86; 95% CI 1.54–2.13; *p* = 0.001), preoperative PVR volume ≥ 250 mL (OR 1.22; 95% CI 1.10–1.32; *p* = 0.02), preoperative catheterization (OR, 0.56; 95% CI 0.34–0.78; *p* = 0.003), increased operation time (OR, 3.87; 95% CI 1.62–4.19; *p* = 0.002), and resected tissue weight ≥ 40 g (OR, 1.032; 95% CI, 1.015–1.048; *p* = 0.002) were found to be independent predictors of TUI. *Conclusions*: The incidence of TUI following HoLEP was found to be 15.96% in patients, with a recovery rate of 78.94% within three months post-surgery. Predictive factors for TUI included age at surgery, prostatic volume, preoperative catheterization, high PVR, longer operative time, and resected tissue weight.

## 1. Introduction

Holmium:YAG laser enucleation of the prostate (HoLEP) is considered one of the most common laser surgical procedures for the treatment of benign prostatic hyperplasia (BPH), as outlined in the European guidelines for non-neurogenic male lower urinary tract symptoms (LUTS) [1,2]. In 1998, the classic three-lobe form of HoLEP received its initial description [3]. Later, the technique underwent several modifications to achieve better outcomes. The outcomes of HoLEP, including urinary flow and patient satisfaction, demonstrate similarities to those of transurethral resection of the prostate (TURP), the conventional therapeutic method for BPH [4]. Furthermore, studies have demonstrated a significant decrease in perioperative morbidity, catheterization time, hospital stay, and reoperation rate compared to TURP [5]. Moreover, the procedure is equally suitable for both small and large prostate glands, even those exceeding 200 g in size, with clinical outcomes comparable to those of robot-assisted simple prostatectomy [6,7]. Therefore, HoLEP is a method that is not dependent on prostate gland size [8]. It has recently been suggested that HoLEP has become the preferred method for surgically treating BPH [9].

While the advantages of HoLEP are numerous, postoperative transient urinary incontinence (TUI) remains a substantial concern after surgery, with the potential to significantly impact the patient’s quality of life (QoL) [10]. Recent reports have indicated that the occurrence of postoperative urinary incontinence (UI) following HoLEP ranges from 1.4% to 43% [11,12]. This significant variation in UI rates is likely due to multiple factors and could be associated with disparities in surgeon expertise, surgical technique, and patient-specific characteristics. Despite the numerous established benefits, the spontaneous resolution of TUI within a three-month timeframe may potentially diminish the possibility of both surgeons and patients utilizing HoLEP [12,13]. Based on our experience, the most common complication linked to HoLEP is postoperative TUI.

Although TUI is a relatively common complication following HoLEP, the majority of existing studies have concentrated on the factors that contribute to stress urinary incontinence (SUI) in the post-HoLEP period [14,15].

This study attempted to identify the incidence and predictors of TUI after HoLEP performed by a single surgeon at a single institution. By identifying these predictors, surgeons can enhance their ability to provide patients with more effective guidance regarding the procedure, thereby facilitating enhanced informed decision-making and increased patient satisfaction.

## 2. Materials and Methods

A retrospective analysis was conducted on a prospectively maintained database of the first 149 sequential HoLEP procedures performed by a single surgeon between June 2022 and December 2023, after approval from the institutional review board (No. 201/02.05.2022). The exclusion criteria included the initial cases that were essential for the surgeon’s learning process (*n* = 20), as well as cases with no postoperative follow-up (*n* = 2). The study eliminated patients (*n* = 7) who had neurological diseases, a history of urethral surgery, or pre-operative incontinence. We also excluded one patient who had prolonged UI up to one year after surgery.

All patients had BPH-related LUTS. In case of prostate cancer suspicion, we performed transrectal ultrasound biopsies at the same time or before HoLEP.

All patients underwent routine preoperative evaluation. The variables considered in this study included age, baseline demographic data, body mass index (BMI), International Prostate Symptom Score (IPSS), quality of life (QoL) index, maximum urinary flow rate (Qmax), and postvoid residual urine (PVR) volume. Abdominal ultrasound was utilized to conduct measurements of prostatic volume and PVR. There were no restrictions on the size of the prostate. The surgical procedures were performed by a single, experienced surgeon. The duration of catheterization, length of hospital stay, and occurrences of adverse events during the peri- or postoperative period were documented. The operative data included the total operative time, encompassing the duration of enucleation and morcellation, as well as the tissue weight (g).

Briefly, we used a three-lobe HoLEP technique. A 100 W MultiPulse HoPlus (Asclepion Laser Technologies GmbH, Jena, Germany) with a 550 μm laser fiber, a Storz 26 French continuous flow Storz resectoscope (Karl Storz, Tuttlingen, Germany), together with a laser bridge adapter, and an integrated morcellation module were used. Laser settings for enucleation were 2 J and 45 Hz.

The mean Qmax rate, postvoid residual (PVR), and IPSS were evaluated at 1, 3, 6, and 12 months following HoLEP. The assessment of continence status was conducted based on the established criteria of the International Continence Society (ICS) and by responding to the question “Do you experience an involuntary urine loss?” [16]. Complete urinary control was considered as no UI. For this study, UI was considered transient if it remitted within six months following the surgical procedure, consistent with previous literature [17]. Long-term UI was defined as any leakage lasting more than six months. The study compared patients with and without TUI based on various pre- and perioperative factors. Patients who experienced UI after surgery were offered anticholinergic and/or beta-3 agonist medications during follow-up visits. The risk of patients was assessed by stratifying them based on their preoperative prostate size (>100 g or ≤100 g), preoperative urine volume (>250 mL or ≤250 mL), and preoperative catheter-dependent status.

Continuous variables were reported as mean and standard deviation, and categorical variables were expressed as frequencies and percentages. The clinical and urodynamic characteristics were evaluated to identify statistically significant differences between the TUI and no TUI groups. Statistical analyses were conducted using SPSS^®^ 27.0 (IBM Corporation, Armonk, NY, USA) with 2-tailed and nonparametric statistics. Fisher’s exact test was employed to compare categorical factors, with an independent samples *t*-test for quality of means, and binary regression for multivariate analyses. Statistical significance was considered at *p* < 0.05.

## 3. Results

The study included 119 patients with BPH. During the second week after surgery, 19 of them experienced postoperative TUI, which accounted for 15.96% of the patients. Of those 19 patients, 15 (78.94%) recovered within three months from the date of the surgery. The prevalence of stress and urge urinary incontinence, as well as postvoid dribbling, was found to be 4.2% in 5 patients, 10.8% in 12 patients, and 5.88% in 7 patients.

Table 1 presents the preoperative baseline characteristics. Patients who developed TUI differed significantly from those who did not in terms of patient age (no TUI 66.4 ± 7.2 vs. TUI 69.7 ± 6.5, *p* < 0.001), prostate volume (no TUI 79.24 ± 20.37 vs. TUI 122.7 ± 28.94, *p* 0.015), catheter dependency (no TUI 15 (15.30%) vs. TUI 11 (57.89%), *p* <0.001), and PVR (no TUI 90.1 ± 64.9 vs. TUI 179.8 ± 145.7, <0.001).

TUI, transient urinary incontinence; BMI, body mass index; PSA, prostate-specific antigen; IPSS, international prostate symptom score; QoL, quality of life; Qmax, maximum flow rate; PVR, postvoid residual.

In terms of the operative results reported in Table 2, patients who developed TUI had a longer total operative time (52.1 ± 36.0 vs. 71.7 ± 50.9, *p* < 0.001) and enucleation time (44.5 ± 14.6 vs. 63.2 ± 17.3, *p* = 0.021), as well as a higher resected prostate weight (28.2 ± 26.8 vs. 52.5 ± 48.4, *p* < 0.001).

There were no differences in the postoperative data illustrated in Table 3, except for post-op IPSS (no TUI 3.8 ± 3.9 vs. TUI 7.5 ± 8.3, *p* = 0.013) and post-op QoL (no TUI 1.1 ± 1.3 vs. TUI 2.7 ± 1.4, *p* = 0.031).

TUI, transient urinary incontinence; Qmax, maximum flow rate; PVR, postvoid residual; IPSS, international prostate symptom score; QoL, quality of life.

In the multivariate regression analysis (Table 4), increased age (odds ratio [OR], 3.47; 95% confidence interval [CI], 1.56~7.78; *p* = 0.002), prostate volume ≥ 100 mL (OR 1.86; 95% CI 1.54–2.13; *p* = 0.001), preoperative PVR volume ≥ 250 mL (OR 1.22; 95% CI 1.10–1.32; *p* = 0.02), preoperative catheterization (OR, 0.56; 95% CI 0.34–0.78; *p* = 0.003), increased operation time (OR, 3.87; 95% CI 1.62–4.19; *p* = 0.002), and resected tissue weight ≥ 40 g (OR, 1.032; 95% CI, 1.015–1.048; *p* = 0.002) were found to be independent predictors of TUI.

## 4. Discussion

TUI is a common postoperative complication of HoLEP, which can cause significant discomfort for both patients and clinicians. Involuntary loss of urine is a hygienic and socially difficult situation that significantly decreases patients’ QoL. Moreover, complaints of incontinence symptoms can be very stressful for clinicians. For young men, QoL is also related to ejaculatory dysfunction and fertility [18]. There is an interest in developing ejaculation-preserving techniques that show promise for improving symptoms, preserving ejaculation, and the quality of the sperm [19,20]

Some authors focused on postoperative TSUI following HoLEP, with rates ranging from 1.4% to 43% [12,13]. In this study, we considered any involuntary urinary leakage as TUI, which included stress or urge urinary incontinence, as well as postvoid dribbling. Our definition of TUI was more comprehensive, as all of these symptoms can significantly impact a patient’s QoL. In this study, 15.96% of patients experienced postoperative TUI, and 78.94% recovered within three months of surgery.

Previous studies that have looked for associations with UI after HoLEP have shown varied results. Several studies examined the impact of surgeon experience and the learning curve on postoperative TUI. Some studies reported higher rates of UI in the first 20 cases [21,22], while others reported higher rates in the first 40–50 cases [23,24]. It is frequently noted that there is an association between UI and the learning curve. This is because, during the learning curve, the operation time is longer, enucleation can be incomplete, and complications are more frequent. To prevent postoperative UI, it is important to have a structured mentorship program to shorten the learning curve [24]. Therefore, we excluded the first 20 cases, which we considered to be part of the surgeon’s learning curve.

This study revealed various predictive factors for the incidence of TUI following HoLEP, such as the patient’s age, prostate volume, preoperative PVR, preoperative catheterization, total operation time, and resected tissue weight.

Increased age may be linked to a greater occurrence of postoperative TUI as a result of the potentially more fragile and sparse sphincteric tissue in older individuals. This can lead to increased susceptibility to tissue damage caused by force. In addition, in the surgical treatment of BPH, an overactive bladder was observed in more than half of the patients over 70 years old [25]. Increased age was significantly associated with TUI in other studies [24,26].

Larger prostate size is correlated with an extended duration of surgery and an increased incidence of excised tissue. An extended duration of surgery results in a prolonged period of sheath manipulation across the external sphincter, potentially resulting in increased sphincter injury. Nevertheless, prostates that are larger and have well-capsulated adenomas may not always necessitate a lengthier duration of surgery [27]. According to Yalcin et al., a shorter time of surgery for the lateral lobe, lower laser energy usage, and better enucleation efficiency are significantly associated with a low transverse length of prostate [27]. Patients with significantly enlarged prostates that obstruct the bladder outlet may have a less well-developed sphincter. The surgery involves radical removal of the adenoma, creating a large prostatic fossa. This can result in urine trapping and leakage, leading to TSUI after HoLEP [28]. Several studies have indicated that TUI is typically a symptomatic urge produced by the healing of the fossa or is linked to detrusor instability secondary to long-standing BPH, urinary tract infections, or thermal lesions of the prostatic capsule caused by holmium laser exposure [12,29]. In order to limit thermal damage, it is recommended to reduce the amount of energy delivered during the apical dissection. The energy delivered may influence the urgency urinary incontinence (UUI) secondary to the healing of the peripheral zone [12]. In addition, UUI is also common in the series of open simple prostatectomies where mechanical enucleation is performed [30].

Patients who underwent preoperative catheterization had higher rates of TUI. This correlation may be due to the fact that these patients are more likely to have severe BPH, which can result in longer endoscopic manipulation and predispose them to TUI [24]. After 11 years of experience with HoLEP, Gild et al. reported that preoperative catheterization is a predictive factor for persistent or recurrent symptoms [31].

The results were replicated in cases where the preoperative PVR was ≥250 mL. Grosso et al. found that preoperative PVR ≥ 250 mL was associated with a risk of unsuccessful outcomes [25]. Similarly, Jaeger et al. conducted a study on patients with chronic urinary retention (PVR > 300 mL) who underwent HoLEP. The study revealed that, although most patients were free of catheters one month after the surgery, the median Q-max remained significantly low. This suggests a potential negative interference of detrusor underactivity in this group. Before surgery, these patients should undergo urodynamic evaluation [32].

The surgical technique used may have an impact on post-operative continence. In this case, the enucleation was performed using the ‘3 lobes technique’ and no early release apical procedure was carried out. The antero-posterior dissection method described by Endo et al. accurately duplicates the open simple prostatectomy operation. This technique enabled a more accurate determination of the mucosal flap located between the apex and sphincter, leading to a reduction in urine incontinence from 25.2% to 2.7% [33]. The removal of BPH tissue between the time frame of 10 to 2 o’clock poses challenges and necessitates careful handling to prevent thermal damage to the sphincter. Saitta et al. described the ‘En Bloc technique of HoLEP’ with early apical release, which was found to be associated with less SUI [34]. This technique offers several advantages, including a shorter operative time, improved visualization of the dissection plane due to less bleeding, and good irrigation. The objective is to improve the efficiency and safety of the enucleation process. Stretching can be achieved more quickly when the external sphincter is attached to one side and the scope is orientated to the opposite side. To prevent complications such as early incontinence, Nevo et al. proposed a partial HoLEP procedure that involves excising only the median lobe. This partial HoLEP was reported to have a shorter operative time and remove less tissue compared to the standard HoLEP. Both groups showed significant improvements in PVR and Qmax from the surgery to three and 12 months [35].

For most patients with SUI, conservative treatment, such as pelvic floor exercises in the first 4 weeks, is often recommended [36]. In cases of UUI or mixed urinary incontinence (MUI) after prostatic surgery at 3 and 6 months, medication with anticholinergic and non-steroidal anti-inflammatory drugs can be effective [37].

Our study has several limitations. First, the number of patients was limited. The study’s retrospective design may have introduced bias, as data collection and patient selection were not randomized. The study was conducted at a single center by a single surgeon, which may limit the generalizability of the findings to other settings and surgeons. Second, the urologist reported the presence of UI during postoperative visits, without quantification, such as a pad test or voiding diary or urodynamic evaluation for the entire series, which could have provided a more comprehensive assessment of incontinence and its impact on patients. Additionally, most cases of UI occurred within the first 6 months. There are no widely accepted standard criteria for reporting UI. In this text, we refer to the ICS definition of UI as a subjective complaint [17]. The definitions of SUI, UUI, and MUI were established through clinical practice. Due to the small size of this subgroup, multivariate regression analysis of predictive factors of SUI, UUI, and MUI was not relevant. Additionally, other factors were not taken into consideration, including the incidence of associated prostate adenocarcinoma, anticoagulation, timing of LUTS, use of preoperative medical therapies, duration of irrigation, early postoperative complications, or reinterventions. Further studies could refine the understanding of TUI predictors by exploring these factors.

Despite these limitations, this study presents a single-surgeon experience with HoLEP and provides important data regarding the incidence and predictors of postoperative TUI. TUI after HoLEP has significant implications for patient QoL, which may be a factor in the hesitance of widespread adoption of HoLEP. Our study demonstrates that TUI disappears in the majority of patients, mostly within the first 3 months. Identifying risk factors that predict TUI may also improve preoperative counseling, reduce patient frustration, and improve patient and physician confidence. The present study highlights the necessity of conducting future prospective randomized and multicentric studies in order to mitigate this problem.

## 5. Conclusions

TUI after HoLEP was observed in 15.96% of patients and 78.94% recovered within three months after surgery. Preoperative counseling of patients regarding expected urinary symptoms after any bladder outlet procedure is advisable. Patients can be informed that age at surgery, prostate volume, preoperative catheterization, high PVR, and prolonged operative time are predictors of TUI. Because the HoLEP technique completely eliminates the adenoma, we suggest that the indication for surgery may be changed to an early stage of the BPH to reduce the impact on micturition physiology. Larger cohorts and multicentric studies that include urodynamic evaluation are needed to validate our findings.

## Figures and Tables

**Table 1:** Baseline characteristics.

Variable	Patients without TUI (*n* = 98)	Patients with TUI (*n* = 19)	*p*-Value
Age (years)	66.4 ± 7.2	69.7 ± 6.5	<0.001
Diabetes (N, %)	12 (12.24%)	3 (15.78%)	0.764
BMI	28.7 ± 7.8	29.6 ± 5.8	0.7031
Serum PSA (ng/mL)	6.84 ± 12.6	6.95 ± 7.3	0.463
Prostate volume (mL)	79.24 ± 20.37	122.7 ± 28.94	0.015
Pre-operative catheterization (N, %)	15 (15.30%)	11 (57.89%)	<0.001
IPSS	21.2 ± 8.4	22.0 ± 7.6	0.784
QoL	4.8 ± 1.7	4.9 ± 1.6	0.618
Qmax (mL/s)	9.78 ± 4.62	11.55 ± 4.23	0.311
PVR (mL)	90.1 ± 64.9	179.8 ± 145.7	<0.001

Values are presented as mean ± standard deviation or number (%).

**Table 2:** Operative data.

Variable	Patients without TUI (*n* = 98)	Patients with TUI (*n* = 19)	*p*-Value
Total operative time (min)	52.1 ± 36.0	71.7 ± 50.9	<0.001
Enucleation time (min)	44.5 ± 14.6	63.2 ± 17.3	0.021
Morcellation time (min)	14.7 ± 16.1	17.3 ± 21.2	0.137
Resected tissue (g)	28.2 ± 26.8	52.5 ± 48.4	<0.001

Values are presented as mean ± standard deviation. TUI, transient urinary incontinence.

**Table 3:** Postoperative data.

Variable	Patients without TUI (*n* = 98)	Patients with TUI (*n* = 19)	*p*-Value
Post-Op Catheterization Time (days)	5.5 ± 3.5	5.9 ± 3.1	0.613
Hospital stay (days)	1.40 ± 1.1	1.38 ± 1.3	0.973
Post-Op Qmax (mL/s)	24.4 ± 17.5	24.2 ± 11.8	0.982
Post-op PVR (mL)	23.4 ± 29.3	23.5 ± 28.5	0.341
Post-Op IPSS	3.8 ± 3.9	7.5 ± 8.3	0.013
Post-Op QoL	1.1 ± 1.3	2.7 ± 1.4	0.031

Values are presented as mean ± standard deviation.

**Table 4:** Univariate and multivariate logistic regression analysis of the predictors of postoperative transient incontinence.

Variable	Univariate Analysis		Multivariate Analysis	
	Odds Ratio (95% CI)	*p*-Value	Odds Ratio (95% CI)	*p*-Value
Age (<65 vs. ≥65), yr	2.84 (1.53–5.21)	0.001	3.47 (1.56–7.78)	0.002
Prostate volume (<100 vs. ≥100), mL	1.74 (1.45–2.17)	0.01	1.86 (1.54–2.13)	0.001
Preoperative PVR volume (<250 vs. ≥250), mL	1.19 (1.11–1.34)	0.01	1.22 (1.10–1.32)	0.02
Pre-operative catheterization	0.48 (0.31–0.75)	0.004	0.56 (0.34–0.78)	0.003
Total operation time (<60 vs. ≥60), min	2.47 (1.24–3.73)	0.005	3.87 (1.62–4.19)	0.002
Resected tissue (<40 vs. ≥40), g	1.013 (1.003–1.023)	0.003	1.032 (1.015–1.048)	0.002

CI: confidence interval, OR: odds ratio.

## Data Availability

The data that support the conclusions of this study will be made available by the authors on reasonable request.
